# Supplementary material for: Distinct miRNA Profile of Cellular and Extracellular Vesicles Released from Chicken Tracheal Cells Following Avian Influenza Virus Infection
Source: Vaccines (Basel). 2020 Aug 5;8(3):438. doi: 10.3390/vaccines8030438 (PMC7565416; doi:10.3390/vaccines8030438)
Supplement: Supplementary file 1 [file vaccines-08-00438-s001.pdf]

SUPPLEMENTARY S1

Supplementary figures.

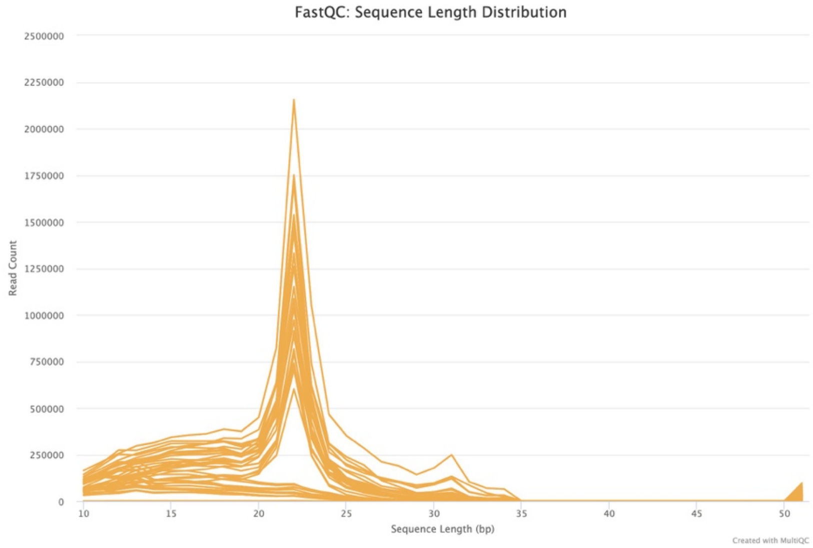

Figure S1. Sequence length distribution of miRNAs. The distribution of fragment sizes (read lengths) were concentrated at 22 nucleotides.

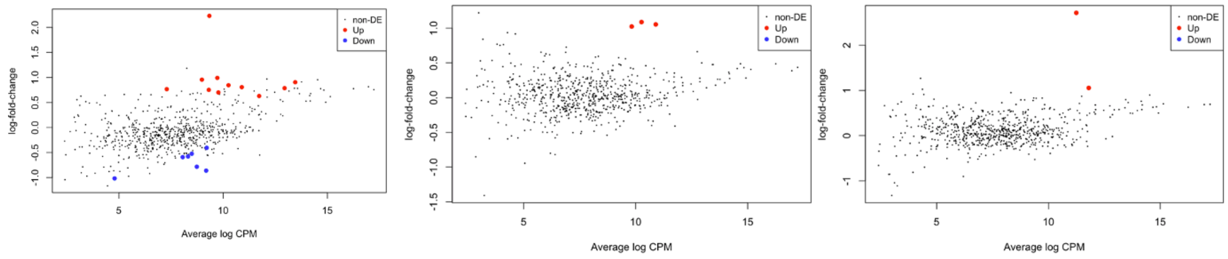

(**a**) (**b**) (**c**)

Figure S2. Up- and down-regulated DE miRNAs in the (a) TOC 3h AIV, (b) TOC 3h LPS and (c) TOC 3h polyI:C treatment groups. A mean-difference plot showing log-intensity ratios versus log-intensity values in TOC samples 3h post-treatment: (**a**) AIV group; (**b**) LPS group; (**c**) polyI:C group.

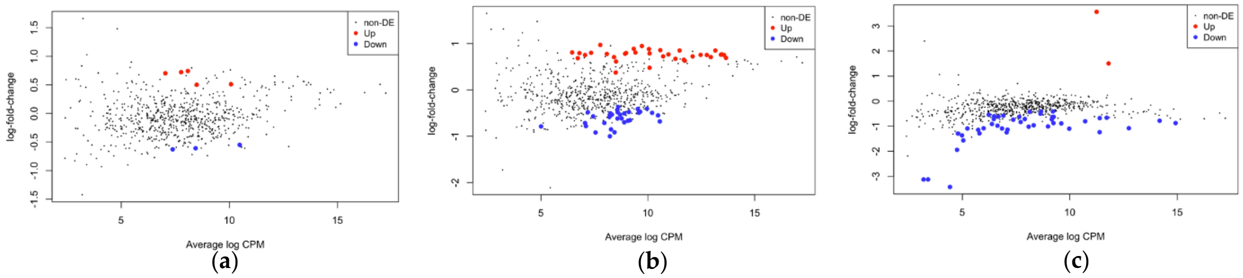

(**a**) (**b**) (**c**)

Figure S3. Up- and down-regulated DE miRNAs in the (a) TOC 18h AIV, (b) TOC 18h LPS and (c) TOC 18h polyI:C treatment groups. A mean-difference plot showing log-intensity ratios versus log-intensity values in TOC samples 18h post-treatment: (**a**) AIV group; (**b**) LPS group; (**c**) polyI:C group.

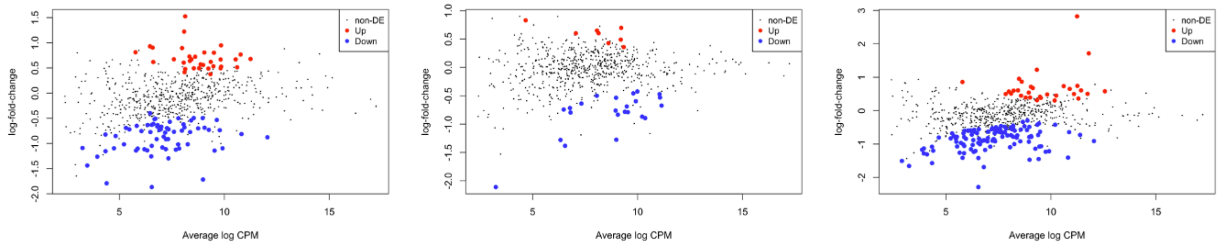

(**a**) (**b**) (**c**)

Figure S4. Up- and down-regulated DE miRNAs in the (a) EV AIV, (b) EV LPS and (c) EV polyI:C treatment groups. A mean-difference plot showing log-intensity ratios versus log-intensity values in EVs: (**a**) AIV group; (**b**) LPS group; (**c**) polyI:C group.

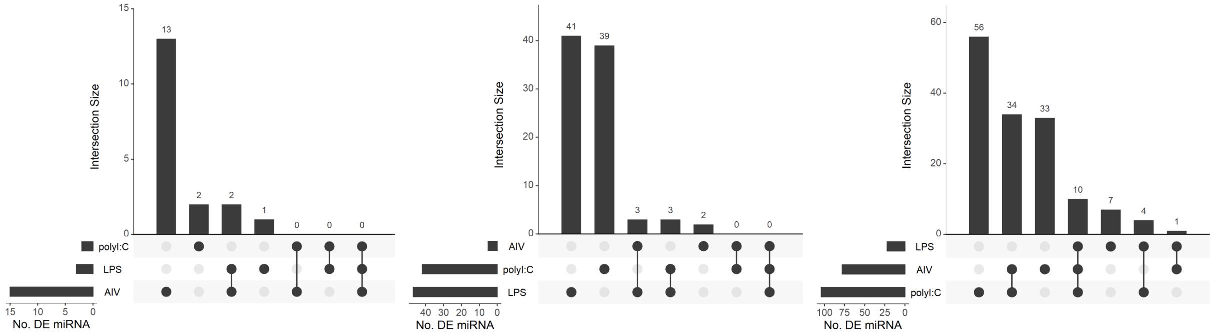

(**a**) (**b**) (**c**)

Figure S5. Visualization of intersecting sets of DE miRNAs in (a) TOC 3h groups treated with AIV, LPS and polyI:C, (b) TOC 18h groups treated with AIV, LPS and polyI:C and (c) EXO groups treated with AIV, LPS and polyI:C. The UpSet intersection diagrams show the number of DE miRNAs (y-axis) that have been detected in each of the intersection sets of treatment groups (x-axis), indicated by the connected points in the lower part of the plot. All intersection sets and lists of DE miRNAs in each intersection set for TOC 3h, TOC 18h and EXO treatment groups are shown in Tables S3, S4 and S5, respectively.

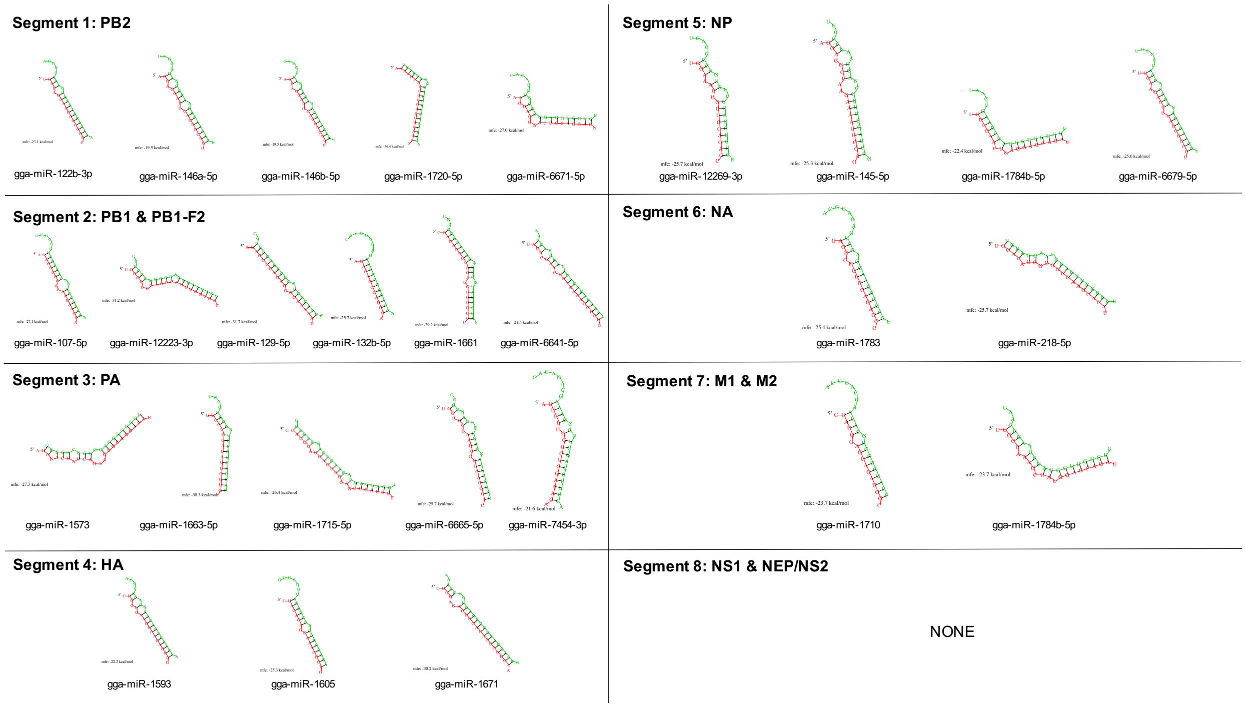

Figure S6. Predicted secondary structures for miRNA targeting viral genome. The secondary structures for the miRNA-RNA interactions were predicted using RNAhybrid, which was also used to predict and confirm the mfe predicted by miRanda.

Supplementary tables.

Table S1. Normalized reads from small RNA sequencing. The number of clean reads by treatment group and a total number of clean reads following Trimmomatic trimming and FastQC quality control treatment of data.

	Treatment group	Number of normalized reads
EV samples	EV AIV	9,075,825
	EV LPS	6,670,361
	EV polyI:C	5,787,236
	EV control	5,764,825
	TOTAL	27,298,247
TOC samples	TOC AIV 3h	32,126,182
	TOC AIV 18h	43,368,495
	TOC LPS 3h	29,199,455
	TOC LPS 18h	23,759,359
	TOC polyI:C 3h	23,598,624
	TOC polyI:C 18h	20,956,379
	TOC CTRL 3h	30,809,730
	TOC CTRL 18h	31,991,864
	TOTAL	235,810,088

Table S2. Intersecting sets of DE miRNAs among TOC 3h groups treated with AIV, LPS and polyI:C. There were 2 miRNAs that were DE in both the TOC 3h AIV and TOC 3h LPS groups. TOC 3h AIV, LPS and polyI:C groups each had 13, 1 and 2 unique DE miRNAs, respectively. In the "Expression" column, green arrows represent up-regulation and red arrows represent down-regulation.

TOC 3h			
Treatment group	Total	MiRNA	Expression
AIV TOC 3h, LPS TOC 3h & polyI:C TOC 3h	0	N/A	N/A
AIV TOC 3h & LPS TOC 3h	2	gga-miR-1608	↑
		gga-miR-6705-5p	↑
AIV TOC 3h & polyI:C TOC 3h	0	N/A	N/A
LPS TOC 3h & polyI:C TOC 3h	0	N/A	N/A
AIV TOC 3h	13	gga-let-7c-5p	↑
		gga-let-7l-5p	↑
		gga-miR-129-5p	↑
		gga-miR-132a-3p	↑
		gga-miR-1434	↑
		gga-miR-1692	↓
		gga-miR-1738	↓
		gga-miR-210a-5p	↑
		gga-miR-221-5p	↑
		gga-miR-383-5p	↓
		gga-miR-425-5p	↑
		gga-miR-455-5p	↑
		gga-miR-6611-5p	↓
LPS TOC 3h	1	gga-miR-6704-5p	↑
polyI:C TOC 3h	2	gga-miR-12235-5p	↑
		gga-miR-12253-5p	↑

Table S3. Intersecting sets of DE miRNAs among TOC 18h groups treated with AIV, LPS and polyI:C. There were 3 miRNAs that were DE in both the TOC 18h AIV and TOC 18h LPS groups. There were 3 miRNAs that were DE in both the TOC 18h LPS and TOC 18h polyI:C groups. TOC 18h AIV, LPS and polyI:C groups each had 2, 41 and 39 unique DE miRNAs, respectively. In the "Expression" column, green arrows represent up-regulation and red arrows represent down-regulation.

TOC 18h			
Treatment group	Total	MiRNA	Expression
AIV TOC 18h, LPS TOC 18h & polyI:C TOC 18h	0	N/A	N/A
AIV TOC 18h & LPS TOC 18h	3	gga-miR-12234-5p	↓
		gga-miR-1451-5p	↑
		gga-miR-1563	↑
AIV TOC 18h & polyI:C TOC 18h	0	N/A	N/A
LPS TOC 18h & polyI:C TOC 18h	3	gga-miR-12228-3p	↓
		gga-miR-2184a-3p	↓
		gga-miR-6569-5p	↓
AIV TOC 18h	2	gga-miR-12244-5p	↑
		gga-miR-1793	↓
LPS TOC 18h	41	gga-let-7b	↑
		gga-let-7c-5p	↑
		gga-let-7l-5p	↑
		gga-miR-10a-5p	↑
		gga-miR-10c-5p	↑
		gga-miR-12239-3p	↓
		gga-miR-12266-5p	↓
		gga-miR-124a-5p	↓
		gga-miR-145-5p	↑
		gga-miR-146a-5p	↑
		gga-miR-146b-5p	↑
		gga-miR-1559-5p	↑
		gga-miR-15b-5p	↑
		gga-miR-15c-5p	↑
		gga-miR-16-5p	↑
		gga-miR-16c-5p	↑
		gga-miR-17-5p	↑
		gga-miR-1701	↓
		gga-miR-1729-5p	↑
		gga-miR-1761	↓
		gga-miR-1783	↓
		gga-miR-1795	↓
		gga-miR-1809	↓
		gga-miR-181a-5p	↑
		gga-miR-181b-5p	↑
		gga-miR-18a-5p	↑
		gga-miR-191-5p	↑
		gga-miR-205a	↑
		gga-miR-210a-5p	↑
		gga-miR-2131-5p	↑
		gga-miR-2184a-5p	↑
		gga-miR-221-5p	↑
		gga-miR-30a-5p	↑
		gga-miR-30b-5p	↑
		gga-miR-30c-5p	↑
		gga-miR-30d	↑
		gga-miR-425-5p	↑
		gga-miR-455-5p	↑
		gga-miR-6602-5p	↓
		gga-miR-6632-5p	↓

		gga-miR-7457-5p	↓
polyI:C TOC 18h	39	gga-let-7f-5p	↓
		gga-let-7g-5p	↓
		gga-miR-12208-5p	↓
		gga-miR-12228-5p	↓
		gga-miR-12235-5p	↑
		gga-miR-12246-5p	↓
		gga-miR-12248-3p	↓
		gga-miR-12248-5p	↓
		gga-miR-12253-5p	↑
		gga-miR-130c-5p	↓
		gga-miR-1465	↓
		gga-miR-1551-5p	↓
		gga-miR-1561	↓
		gga-miR-1593	↓
		gga-miR-1656	↓
		gga-miR-1671	↓
		gga-miR-1676-5p	↓
		gga-miR-1692	↓
		gga-miR-1720-5p	↓
		gga-miR-1735	↓
		gga-miR-1750	↓
		gga-miR-1751-5p	↓
		gga-miR-1784b-3p	↓
		gga-miR-190a-5p	↓
		gga-miR-196-5p	↓
		gga-miR-1c	↓
		gga-miR-216c	↓
		gga-miR-365-2-5p	↓
		gga-miR-449a	↓
		gga-miR-499-5p	↓
		gga-miR-6563-5p	↓
		gga-miR-6564-5p	↓
		gga-miR-6611-5p	↓
		gga-miR-6641-5p	↓
		gga-miR-6706-5p	↓
		gga-miR-7	↓
		gga-miR-7458-5p	↓
		gga-miR-7470-5p	↓
		gga-miR-7480-5p	↓

Table S4. Intersecting sets of DE miRNAs among EV groups treated with AIV, LPS and polyI:C. There were 10 miRNAs that were DE in all EV groups. In addition, there was 1 miRNA that was DE in both the EV AIV and EV LPS groups. There were 34 DE miRNAs that were DE in both the EV AIV and EV polyI:C groups and 4 miRNAs that were DE in both EV LPS and EV polyI:C groups. EV AIV, LPS and polyI:C groups each had 33, 7 and 56 unique DE miRNAs, respectively. In the "Expression" column, green arrows represent up-regulation and red arrows represent down-regulation.

	EV		
Treatment group	Total	MiRNA	Expression
EV AIV, EV LPS & EV polyI:C	10	gga-miR-107-5p	↓
		gga-miR-12284-3p	↓
		gga-miR-1464	↓
		gga-miR-1727	↓
		gga-miR-1784b-5p	↓
		gga-miR-205b	↓
		gga-miR-210a-5p	↓
		gga-miR-449b-5p	↓
		gga-miR-6665-5p	↓
		gga-miR-7482-5p	↓
EV AIV & EV LPS	1	gga-miR-383-5p	↑
EV AIV & EV polyI:C	34	gga-miR-12247-3p	↓
		gga-miR-12252-3p	↑
		gga-miR-12253-5p	↑
		gga-miR-12254-5p	↓
		gga-miR-12260-5p	↓
		gga-miR-12269-3p	↓
		gga-miR-1306-5p	↓
		gga-miR-142-5p	↓
		gga-miR-1553-5p	↓
		gga-miR-1605	↓
		gga-miR-1626-5p	↓
		gga-miR-1632-5p	↓
		gga-miR-1670	↑
		gga-miR-1677-5p	↓
		gga-miR-1715-5p	↓
		gga-miR-1722-5p	↓
		gga-miR-1730-5p	↓
		gga-miR-216a	↓
		gga-miR-218-5p	↓
		gga-miR-2184b-5p	↓
		gga-miR-301b-5p	↓
		gga-miR-3532-5p	↓
		gga-miR-3536	↓
		gga-miR-365b-5p	↓
		gga-miR-6516-5p	↓
		gga-miR-6550-5p	↓
		gga-miR-6596-5p	↓
		gga-miR-6604-5p	↓
		gga-miR-6606-5p	↑
		gga-miR-6639-5p	↓
		gga-miR-6671-5p	↓
		gga-miR-6675-5p	↓
		gga-miR-7454-3p	↓
		gga-miR-7456-5p	↓
EV LPS & EV polyI:C	4	gga-miR-12223-3p	↓
		gga-miR-132b-5p	↓
		gga-miR-1597-5p	↓
		gga-miR-211	↓

AIV EV	33	gga-miR-122-5p	↑
		gga-miR-12221-5p	↑
		gga-miR-12229-5p	↑
		gga-miR-12244-5p	↓
		gga-miR-122b-3p	↓
		gga-miR-128-1-5p	↓
		gga-miR-1452	↑
		gga-miR-1563	↑
		gga-miR-1575	↑
		gga-miR-15b-5p	↑
		gga-miR-1637	↑
		gga-miR-1648-5p	↓
		gga-miR-1651-5p	↓
		gga-miR-1661	↓
		gga-miR-1664-5p	↓
		gga-miR-1710	↓
		gga-miR-1724	↓
		gga-miR-1784-5p	↓
		gga-miR-1801	↓
		gga-miR-1815	↓
		gga-miR-193a-5p	↑
		gga-miR-194	↑
		gga-miR-301a-5p	↑
		gga-miR-3528	↓
		gga-miR-365-1-5p	↑
		gga-miR-6543-5p	↑
		gga-miR-6590-5p	↓
		gga-miR-6616-5p	↑
		gga-miR-6684-5p	↓
		gga-miR-6708-5p	↑
		gga-miR-726-3p	↓
		gga-miR-7464-3p	↓
		gga-miR-92-5p	↑
EV LPS	7	gga-miR-12272-3p	↑
		gga-miR-12273-5p	↓
		gga-miR-1584	↓
		gga-miR-1618-5p	↑
		gga-miR-3535	↑
		gga-miR-6557-5p	↓
		gga-miR-6697-5p	↓
EV polyI:C	56	gga-miR-12209-3p	↓
		gga-miR-12219-3p	↓
		gga-miR-12235-5p	↑
		gga-miR-12266-5p	↓
		gga-miR-12274-3p	↓
		gga-miR-12274-5p	↓
		gga-miR-12279-3p	↓
		gga-miR-12290-5p	↑
		gga-miR-12295-5p	↑
		gga-miR-1397-5p	↑
		gga-miR-1456-5p	↑
		gga-miR-1462-5p	↓
		gga-miR-1465	↓
		gga-miR-1573	↓
		gga-miR-1598	↓
		gga-miR-1608	↑
		gga-miR-1638	↓
		gga-miR-1649-5p	↑
		gga-miR-1658-5p	↓

	gga-miR-1663-5p	↓
	gga-miR-1667-5p	↓
	gga-miR-1690-5p	↓
	gga-miR-1755	↓
	gga-miR-1775-5p	↓
	gga-miR-1777	↑
	gga-miR-1795	↓
	gga-miR-1802	↓
	gga-miR-1805-5p	↓
	gga-miR-1814	↓
	gga-miR-19a-5p	↓
	gga-miR-204	↓
	gga-miR-210b-5p	↓
	gga-miR-212-5p	↓
	gga-miR-216b	↓
	gga-miR-219a	↓
	gga-miR-23b-5p	↓
	gga-miR-302b-5p	↓
	gga-miR-30b-5p	↓
	gga-miR-449a	↓
	gga-miR-449d-5p	↓
	gga-miR-490-5p	↓
	gga-miR-6559-5p	↓
	gga-miR-6566-5p	↓
	gga-miR-6567-5p	↓
	gga-miR-6582-5p	↓
	gga-miR-6593-5p	↑
	gga-miR-6598-5p	↓
	gga-miR-6669-5p	↓
	gga-miR-6679-5p	↓
	gga-miR-6707-5p	↓
	gga-miR-726-5p	↓
	gga-miR-7444-5p	↓
	gga-miR-7451-5p	↓
	gga-miR-7471-5p	↑
	gga-miR-7473-5p	↓
	gga-miR-7479-5p	↓

Table S5. Pathway analysis summary. Host target genes for DE miRNAs were predicted using the miRDB *Gallus gallus* database. Pathway analysis was then performed using the gene set characterization pipeline by KnowEnG. Only hits with scores greater than or equal to 95 (miRDB score≥95) were considered.

	Total DE			Up-regulated			Down-regulated		
	miRNAs	Target genes	Target pathways	miRNAs	Target genes	Target pathways	miRNAs	Target genes	Target pathways
TOC 3h AIV	15	115	9	11	103	6	4	12	3
TOC 3h LPS	3	39	8	3	39	8	0	N/A	N/A
TOC 3h polyI:C	2	170	5	2	170	5	0	N/A	N/A
TOC 18h AIV	5	28	15	3	17	14	2	11	2
TOC 18h LPS	47	650	17	32	505	8	15	123	10
TOC 18h polyI:C	42	435	6	2	165	6	40	265	1
EV AIV	78	559	18	21	132	8	57	403	10
EV LPS	22	170	11	5	20	3	17	150	9
EV polyI:C	104	929	14	14	188	11	90	715	5

Table S6. Prediction of pathways targeted by up-regulated cellular DE miRNAs following treatment with AIV, LPS and polyI:C. Pathways predicted for up-regulated cellular DE miRNAs collected 3h and 18h post-stimulation following treatment with AIV, LPS and polyI:C using the miRDB chicken database.

		Pathway	miRNA(s)
TOC 3h	TOC 3h AIV	Gene expression (Transcription)	gga-let-7l-5p, gga-miR-129-5p
		R-GGA-212436	gga-let-7c-5p, gga-let-7l-5p, gga-miR-129-5p
		Generic Transcription Pathway	gga-miR-129-5p, gga-miR-6705-5p
		Reversible hydration of carbon dioxide	gga-let-7c-5p, gga-let-7l-5p
		Hemostasis	gga-miR-129-5p, gga-miR-455-5p
		Factors involved in megakaryocyte development and platelet production	gga-miR-455-5p
	TOC 3h LPS	Membrane Trafficking	gga-miR-1608, gga-miR-6704-5p
		R-GGA-199991	gga-miR-1608, gga-miR-6704-5p
		Clathrin-mediated endocytosis	gga-miR-1608
		Cargo recognition for clathrin-mediated endocytosis	gga-miR-1608
		Axon guidance	gga-miR-6704-5p
		CRMPs in Sema3A signaling	gga-miR-6704-5p
		R-GGA-399954	gga-miR-6704-5p
		Neuronal System	gga-miR-1608
	TOC 3h polyI:C	Cardiac conduction	gga-miR-12235-5p
		Muscle contraction	gga-miR-12235-5p
		Neuronal System	gga-miR-12235-5p
		Phase 0 - rapid depolarisation	gga-miR-12235-5p
		R-GGA-352230	gga-miR-12235-5p
TOC 18h	TOC 18h AIV	Collagen formation	gga-miR-1563
		Collagen biosynthesis and modifying enzymes	gga-miR-1563
		Assembly of collagen fibrils and other multimeric structures	gga-miR-1563
		Extracellular matrix organization	gga-miR-1563
		Phase 0 - rapid depolarisation	gga-miR-12244-5p
		Phase 2 - plateau phase	gga-miR-12244-5p

	Cardiac conduction	gga-miR-12244-5p
	R-GGA-381676	gga-miR-12244-5p
	Integration of energy metabolism	gga-miR-12244-5p
	Cell-cell junction organization	gga-miR-12244-5p
	Adherens junctions interactions	gga-miR-12244-5p
	Cell junction organization	gga-miR-12244-5p
	Cell-Cell communication	gga-miR-12244-5p
	Muscle contraction	gga-miR-12244-5p
TOC 18h LPS	R-GGA-212436	gga-let-7b, gga-let-7c-5p, gga-let-7l-5p, gga-miR-17-5p, gga-miR-18a-5p
	Gene expression (Transcription)	gga-let-7l-5p, gga-miR-15b-5p, gga-miR-15c-5p, gga-miR-16-5p, gga-miR-16c-5p, gga-miR-17-5p, gga-miR-18a-5p, gga-miR-30a-5p, gga-miR-30b-5p, gga-miR-30c-5p, gga-miR-30d
	Protein-protein interactions at synapses	gga-miR-145-5p, gga-miR-181a-5p, gga-miR-181b-5p, gga-miR-2131-5p, gga-miR-30a-5p, gga-miR-30b-5p, gga-miR-30c-5p, gga-miR-30d
	Developmental Biology	gga-miR-15b-5p, gga-miR-15c-5p, gga-miR-16-5p, gga-miR-16c-5p, gga-miR-2184a-5p, gga-miR-30a-5p, gga-miR-30b-5p, gga-miR-30c-5p, gga-miR-30d
	Generic Transcription Pathway	gga-miR-18a-5p, gga-miR-181a-5p, gga-miR-181b-5p
	Interactions of neurexins and neuroligins at synapses	gga-miR-145-5p, gga-miR-2131-5p, gga-miR-30a-5p, gga-miR-30b-5p, gga-miR-30c-5p, gga-miR-30d
	Neuronal System	gga-miR-145-5p, gga-miR-181a-5p, gga-miR-181b-5p, gga-miR-2131-5p
	Axon guidance	gga-miR-15b-5p, gga-miR-15c-5p, gga-miR-16-5p, gga-miR-16c-5p
TOC 18h polyI:C	Cardiac conduction	gga-miR-12235-5p
	Muscle contraction	gga-miR-12235-5p
	Neuronal System	gga-miR-12235-5p
	Phase 0 - rapid depolarisation	gga-miR-12235-5p

Table S7. Prediction of pathways targeted by down-regulated cellular DE miRNAs following treatment with AIV, LPS and polyI:C. Pathways predicted for down-regulated cellular DE miRNAs collected 3h and 18h post-stimulation following treatment with AIV, LPS and polyI:C using the miRDB chicken database.

		Pathway	miRNA(s)
TOC 3h	TOC 3h AIV	Metabolism of water-soluble vitamins and cofactors	gga-miR-6611-5p
		Metabolism of vitamins and cofactors	gga-miR-6611-5p
		Nicotinate metabolism	gga-miR-6611-5p
	TOC 3h LPS	N/A	N/A
	TOC 3h polyI:C	N/A	N/A
TOC 18h	TOC 18h AIV	Invadopodia formation	gga-miR-12234-5p
		Extracellular matrix organization	gga-miR-12234-5p
	TOC 18h LPS	Neuronal System	gga-miR-12239-3p, gga-miR-7457-5p
		Post-translational protein modification	gga-miR-124a-5p, gga-miR-1701
		Neddylation	gga-miR-12239-3p, gga-miR-12266-5p
		Degradation of the extracellular matrix	gga-miR-12239-3p
		Metabolism of proteins	gga-miR-124a-5p, gga-miR-1701
		Extracellular matrix organization	gga-miR-12234-5p, gga-miR-12239-3p, gga-miR-2184a-5p
		Ion channel transport	gga-miR-124a-5p
	TOC 18h polyI:C	Neuronal System	gga-miR-12228-5p, gga-miR-1465, gga-miR-1784b-3p, gga-miR-216c, gga-miR-449a, gga-miR-7, gga-miR-7480-5p

Table S8. Prediction of pathways targeted by up-regulated EV DE miRNAs following treatment with AIV, LPS and polyI:C. Pathways predicted for up-regulated EV DE miRNAs collected 3h and 18h post-stimulation following treatment with AIV, LPS and polyI:C using the miRDB chicken database.

	Pathway	miRNA(s)
EV AIV	Post-translational protein modification	gga-miR-15b-5p, gga-miR-301a-5p, gga-miR-6616-5p
	R-GGA-1227986	gga-miR-1575, gga-miR-194, gga-miR-365-1-5p, gga-miR-6616-5p, gga-miR-92-5p
	Axon guidance	gga-miR-15b-5p, gga-miR-1575
	Developmental Biology	gga-miR-15b-5p, gga-miR-1575
EV LPS	Degradation of the extracellular matrix	gga-miR-12272-3p
	Activation of Matrix Metalloproteinases	gga-miR-12272-3p
	Extracellular matrix organization	gga-miR-12272-3p
EV polyI:C	Neuronal System	gga-miR-12235-5p, gga-miR-1456-5p, gga-miR-1608
	Cardiac conduction	gga-miR-12235-5p
	Muscle contraction	gga-miR-12235-5p
	Cargo recognition for clathrin-mediated endocytosis	gga-miR-1456-5p, gga-miR-1608
	Clathrin-mediated endocytosis	gga-miR-1456-5p, gga-miR-1608
	Generic Transcription Pathway	gga-miR-1649-5p
	Potassium Channels	gga-miR-1608
	Voltage gated Potassium channels	gga-miR-1608
	R-GGA-352230	gga-miR-12235-5p
	Bicarbonate transporters	gga-miR-12235-5p

Table S9. Prediction of pathways targeted by down-regulated EV DE miRNAs following treatment with AIV, LPS and polyI:C. Pathways predicted for down-regulated EV DE miRNAs collected 3h and 18h post-stimulation following treatment with AIV, LPS and polyI:C using the miRDB chicken database.

	Pathway	miRNA(s)
EV AIV	Neuronal System	gga-miR-1306-5p, gga-miR-1632-5p, gga-miR-218-5p, gga-miR-449b-5p, gga-miR-6639-5p, gga-miR-7464-3p
	Membrane Trafficking	gga-miR-1464, gga-miR-1626-5p, gga-miR-205b, gga-miR-210a-5p, gga-miR-6639-5p, gga-miR-726-3p
	Adherens junctions interactions	gga-miR-107-5p, gga-miR-12244-5p, gga-miR-6684-5p
	Cell-cell junction organization	gga-miR-107-5p, gga-miR-12244-5p, gga-miR-6684-5p
	R-GGA-199991	gga-miR-1464, gga-miR-1626-5p, gga-miR-205b, gga-miR-210a-5p, gga-miR-6639-5p, gga-miR-726-3p
	Cell junction organization	gga-miR-107-5p, gga-miR-12244-5p, gga-miR-6684-5p
	Cell-Cell communication	gga-miR-107-5p, gga-miR-12244-5p, gga-miR-6684-5p
	R-GGA-112308	gga-miR-1306-5p, gga-miR-449b-5p
EV LPS	Gene expression (Transcription)	gga-miR-12223-3p, gga-miR-1597-5p, gga-miR-1727
	Membrane Trafficking	gga-miR-1464, gga-miR-205b, gga-miR-210a-5p
	R-GGA-212436	gga-miR-1597-5p, gga-miR-1727
	R-GGA-199991	gga-miR-1464, gga-miR-205b, gga-miR-210a-5p
	Clathrin-mediated endocytosis	gga-miR-1464, gga-miR-205b
	R-GGA-194840	gga-miR-211
	Rho GTPase cycle	gga-miR-211, gga-miR-6665-5p
	Generic Transcription Pathway	gga-miR-1597-5p, gga-miR-449b-5p
	R-GGA-1227986	gga-miR-132b-5p, gga-miR-205b, gga-miR-211, gga-miR-449b-5p
EV polyI:C	Gene expression (Transcription)	gga-miR-12223-3p, gga-miR-12274-5p, gga-miR-1597-5p, gga-miR-1727, gga-miR-1755, gga-miR-1775-5p, gga-miR-30b-5p, gga-miR-3536, gga-miR-365b-5p, gga-miR-6550-5p
	Generic Transcription Pathway	gga-miR-1597-5p, gga-miR-1755, gga-miR-1775-5p, gga-miR-3536 gga-miR-365b-5p, gga-miR-449a, gga-miR-449b-5p, gga-miR-449d-5p, gga-miR-6550-5p
	R-GGA-212436	gga-miR-12274-5p, gga-miR-1597-5p, gga-miR-1727, gga-miR-1755, gga-miR-1775-5p, gga-miR-3536, gga-miR-365b-5p, gga-miR-6550-5p
	Neuronal System	gga-miR-1306-5p, gga-miR-1462-5p, gga-miR-1465, gga-miR-1573, gga-miR-1632-5p, gga-miR-1775-5p, gga-miR-19a-5p, gga-miR-218-5p, gga-miR-449a, gga-miR-449b-5p, gga-miR-449d-5p, gga-miR-6639-5p
	Membrane Trafficking	gga-miR-1464, gga-miR-1626-5p, gga-miR-205b, gga-miR-210a-5p, gga-miR-6639-5p
